# 1325. Neurodevelopmental outcomes in preschoolers following viral meningitis is Canterbury, New Zealand

**DOI:** 10.1093/ofid/ofad500.1163

**Published:** 2023-11-27

**Authors:** Natalie G Martin, Jonathan Williman, Tony Walls, Manish Sadarangani, Cameron Grant

**Affiliations:** Christchurch Hospital, Christchurch, New Zealand, Christchurch, Canterbury, New Zealand; University of Otago, Christchurch, New Zealand, Christchurch, Canterbury, New Zealand; University of Otago, Christchurch, Christchurch, Canterbury, New Zealand; University of British Columbia, Vancouver, British Columbia, Canada; University of Auckland, Auckland, Auckland, New Zealand

## Abstract

**Background:**

Most childhood meningitis nowadays is caused by viruses in countries with widespread use of conjugate vaccines. There is limited knowledge about outcomes following childhood viral meningitis, necessary to inform clinical guidelines.

**Methods:**

Children aged 19-42 months in Canterbury New Zealand, who had enterovirus (EV) or human parechovirus (HPEV) meningitis as infants were included. Clinical data was collected. Comprehensive neurodevelopmental assessments were completed using the Bayley Scale for Infant Development (BSID) Third Edition. Mean composite and scaled scores, and proportion below cutoff were assessed in each domain. Normative mean composite scores for any domain is 100. Expected scaled score range is 8-12. Mean scores between EV and HPEV meningitis were compared by t-tests, with P< 0.05 considered significant.

**Results:**

BSID assessments were completed for 33 children (55% male) of mean age 31 months, from 2019-22, including 23 with EV and 10 with HPEV meningitis. There were 24 children of NZ European, 4 Māori, 4 Asian, and 1 Middle Eastern ethnicity. Mean age at diagnosis was 43 days, and mean length of admission was 2.79 days (CI 1.74-3.15). Of 33 children, 32 (97%) received intravenous or intramuscular antibiotics, 6 (18%) received a fluid bolus on arrival at hospital, and 9 (27%) required high dependency or intensive care. Following viral meningitis, parents reported developmental speech concerns in 6 children, and delayed motor milestones in 1 child. There was no reported sensorineural hearing loss. BSID mean composite scores were in expected range for cognition 102 (CI 98-106), language 96 (93-100) and motor 102 (98-106) domains. Mean scaled scores were in expected range for receptive language 9 (9-10), expressive language 9 (9-10), fine motor 11 (11-12) and gross motor 9 (9-10) domains. Overall, 12/33 (36%) children had below expected scores in one area of development including cognition (2/33; 6%), receptive language (6/33; 18%), expressive language (5/33; 15%), and gross motor (6/33; 18%). There were no significant differences between BSID scores in EV and HPEV meningitis.

**Conclusion:**

Following viral meningitis, 36% percent of preschool children had findings below expected in at least one developmental domain, suggesting targeted follow-up should be considered.

**Disclosures:**

**Manish Sadarangani, BM BCh, FRCPC, DPhil**, GlaxoSmithKline: Grant/Research Support|Merck: Grant/Research Support|Moderna: Grant/Research Support|Pfizer: Grant/Research Support|Sanofi Pasteur: Grant/Research Support|Seqirus: Grant/Research Support|Symvivo: Grant/Research Support|VBI Vaccines: Grant/Research Support

